# Aberrant DNA Methylation-Mediated FOXF2 Dysregulation Is a Prognostic Risk Factor for Gastric Cancer

**DOI:** 10.3389/fmolb.2021.645470

**Published:** 2021-09-10

**Authors:** Cheng Zhang, Yong-Zhi Li, Dong-Qiu Dai

**Affiliations:** Department of Gastrointestinal Surgery, The Fourth Affiliated Hospital of China Medical University, Shenyang, China

**Keywords:** gastric cancer, Foxf2, prognosis, DNA methylation, miRNA

## Abstract

**Background:** The prognosis of gastric cancer (GC) patients is poor. The effect of aberrant DNA methylation on FOXF2 expression and the prognostic role of FOXF2 methylation in GC have not yet been identified.

**Methods:** The RNA-Seq and gene methylation HM450 profile data were used for analyzing FOXF2 expression in GC and its association with methylation level. Bisulfite sequencing PCR (BSP) was performed to measure the methylation level of the FOXF2 promoter region in GC cell lines and normal GES-1 cells. The cells were treated with the demethylation reagent 5-Aza-dC, and the mRNA and protein expression levels of FOXF2 were then measured by qRT-PCR and western blot assays. The risk score system from SurvivalMeth was calculated by integrating the methylation level of the cg locus and the corresponding Cox regression coefficient.

**Results:** FOXF2 was significantly downregulated in GC cells and tissues. On the basis of RNA-Seq and Illumina methylation 450 data, FOXF2 expression was significantly negatively correlated with the FOXF2 methylation level (Pearson’s R = −0.42, *p* < 2.2e^−16^). The FOXF2 methylation level in the high FOXF2 expression group was lower than that in the low FOXF2 expression group. The BSP assay indicated that the methylation level of the FOXF2 promoter region in GC cell lines was higher than that in GES-1 cells. The qRT-PCR and western blot assay showed that FOXF2 mRNA and protein levels were increased in GC cells following treatment with 5-Aza-Dc. The methylation risk score model indicated that patients in the high risk group had poorer survival probability than those in the low risk group (HR = 1.84 (1.11–3.07) and *p* = 0.0068). FOXF2 also had a close transcriptional regulation network with four miRNAs and their corresponding target genes. Functional enrichment analysis of the target genes revealed that these genes were significantly related to several important signaling pathways.

**Conclusion:** FOXF2 was downregulated due to aberrant DNA methylation in GC, and the degree of methylation in the promoter region of FOXF2 was related to the prognosis of patients. The FOXF2/miRNAs/target genes axis may play a vital biological regulation role in GC.

## Introduction

Gastric cancer (GC) remains an important cancer worldwide, with a large number of new cases and deaths each year, making it the fifth most frequently diagnosed cancer and the third leading cause of cancer-related deaths (Bray et al., 2018). As the main risk factor for GC, approximately 90% of new GC cases are attributed to *Helicobacter pylori* infection (Plummer et al., 2015). Other risk factors include high salt diet, low-fruit diet, drinking, and smoking (IARC, 2012). Although the morbidity and mortality of GC are slowly decreasing due to advances in the diagnosis and treatment of GC in recent years (Zhang et al., 2017), the 5-years survival rate of patients with GC is still below 29% because of the high invasiveness and recurrence of GC (Allemani et al., 2015). Therefore, the study of molecular mechanisms underlying the development and progression of GC is particularly important to establish novel approaches for treating GC.

Epigenetic dysregulation plays an indispensable role in the development and progression of GC (Ebrahimi et al., 2020). In addition to changes and mutations in the genomic DNA sequence, epigenetic changes are observed in the mechanisms of gene expression regulation, including DNA methylation, chromatin remodeling, alteration in noncoding RNA expression, and histone post-translational modifications, and these changes are reversible (Puneet et al., 2018). Aberrant methylation of the CpG island in the promoter region of the gene plays an important role in its inactivation. Abnormal methylation of the CpG island in the promoter region of the tumor suppressor gene leads to its transcriptional inhibition, downregulation, or deletion of expression, resulting in the development of GC. Thus, understanding the mechanism of epigenetic changes is essential for the diagnosis, treatment, and prevention of GC (Sonohara et al., 2017).

Forkhead box F2 (FOXF2) is a member of the Forkhead box family of transcription factors, and all FOX proteins can bind to DNA. These transcription factors (TFs) act as an activator or inhibitor of gene transcription with a highly conserved 110-amino-acid DNA-binding domain (Coffer and Burgering, 2004; Katoh and Katoh, 2004). FOXF2 is closely related to the regulation of human growth and development and tumors (Aitola et al., 2000; Coffer and Burgering, 2004). Several studies have shown that FOXF2 is a potential tumor suppressor, and it can inhibit epithelial-mesenchymal transition (EMT) and metastasis in breast cancer (Wang et al., 2015). However, the comprehensive upstream and downstream transcriptional regulation roles of FOXF2 in GC remain unclear.

## Materials and Methods

### RNA Sequencing Data Collection and Processing

The Cancer Genome Atlas (TCGA, https://portal.gdc.cancer.gov/), Genotype-Tissue Expression (GTEx, https://www.gtexportal.org/home/), and The Human Protein Atlas (HPA, https://www.proteinatlas.org/) were used to assess the FOXF2 expression level in various tumor tissues, including GC, and multiple human normal tissues. Expression profiling by array from Gene Expression Omnibus (GEO) was also used to confirm FOXF2 expression in GC. The RNA-Seq data of GC generated from the TCGA database were downloaded using the TCGAbiolinks package (Colaprico et al., 2016) in R and used to identify differentially expressed genes (DEGs) in GC and normal gastric tissues. The cutoff criteria were fold change |FC| > 2.0 and FDR <0.05. The Trimmed Mean of M values (TMM) method (Robinson and Oshlack, 2010) was used to normalize the observed counts, and the limma package (Ritchie et al., 2015) was then used for identification with R software.

### GC Cell Lines and Clinical Samples

Human GC cell lines SGC-7901, BGC-823, MGC-803, and MKN-45 and the normal gastric epithelium cell line (GES-1) used in the present study were purchased from the Chinese Academy of Sciences (Shanghai, China). All cell lines were maintained in DMEM medium (Gibco, Germany) containing 10% fetal bovine serum (FBS, MilliporeSigma, Shanghai, China), 100 μg/ml streptomycin (Thermo Fisher Scientific, United States), and 100 U/mL penicillin (Thermo Fisher Scientific) in an incubator with 5% CO_2_ at 37°C. Medium changes were performed every 2–3 days (after reaching 70–80% cell confluence).

Clinical samples including 25 paired gastric tumor tissues and adjacent nontumor tissues (at least 5 cm away from the clear edge of tumor tissues) were collected from patients with GC during surgery in the Fourth Affiliated Hospital of China Medical University. None of the patients received any adjuvant treatments before surgery. The ethical approval for this study was obtained from the Fourth Affiliated Hospital of China Medical University, and written informed consents were signed by all patients. All the tissues were removed during the surgery and immediately stored at −80°C.

### RNA Extraction and Real-Time PCR

Total RNA was extracted from cells and tissues by using TRIzol reagent (Invitrogen, United States), and the concentration and purity of RNA were measured with a spectrophotometer. Total RNA was converted into cDNA by reverse transcription using the PrimeScript™ RT Reagent Kit with gDNA Eraser (TaKaRa, Dalian, China). Quantitative real-time PCR (qRT-PCR) was performed using TB Green® Premix Ex Taq™ II (TaKaRa, Dalian, China). Glyceraldehyde-3-phosphate dehydrogenase (GAPDH) was used as a reference. The primers used for qRT-PCR are shown in Supplementary Table S1. All steps were performed in accordance with the instructions of the reagent manufacturer.

### Bisulfite Sequencing PCR (BSP) and Methylation Drug Treatment

Methprimer 2.0 (Li and Dahiya, 2002) was used to determine whether there are CpG islands in the promoter sequence of FOXF2 and for designing BSP primers. The MiniBEST Universal Genomic DNA Extraction Kit (TaKaRa, Dalian, China) was used to extract DNA from cells and tissues. The EZ DNA Methylation-Gold kit (Zymo Research, Irvine, CA, United States) was used for methylation to modify DNA. The bisulfite-modified DNA was used for BSP. The primers sequences are shown in Supplementary Table S1. Three GC cell lines, namely SGC-7901, MGC-803, and MKN-45, and GES-1 were cultured and treated with 5-Aza-dC (Sigma-Aldrich) at the dose of 15 μmol/L for 3 days. The mRNA and protein expression levels of FOXF2 were measured.

### Cytoplasm and Nucleus Separation

The Nuclear and Cytoplasmic Protein Extraction Kit (Wanleibio, China) was used to isolate cytoplasm and nucleus protein according to the manufacturer’s instructions.

### Western Blotting

The protocols for western blotting were described in a previous study (Zhang et al., 2019). The primary antibodies were incubated at 4°C overnight according to the following dilution ratio: FOXF2 (1:200; Abcam, Cambridge, MA, United States). β-actin and Histone H3 were used as endogenous loading control.

### FOXF2 Methylation and Prognosis Risk Analysis

The Stomach Adenocarcinoma methylation (HM450) profile, downloaded from cBioPortal (Cerami et al., 2012) (http://www.cbioportal.org/), was used to assess correlation between FOXF2 expression and methylation level. The cg locus located at the FOXF2 promoter region was identified using the MEXPRESS tool (Koch et al., 2019), and the association between the methylation level of the target cg locus and FOXF2 expression was then evaluated. SurvivalMeth (Zhang et al., 2020) (http://bio-bigdata.hrbmu.edu.cn/survivalmeth), a web server to investigate the effect of DNA methylation-related functional elements on prognosis, was used to analyze methylation levels and the correlation between methylation and risk score, gene expression, and prognosis. All steps were performed in accordance with the developer’s instructions. The risk score system from SurvivalMeth was calculated by integrating the methylation level of the cg locus and the corresponding Cox regression coefficient. The optimal cutoff value of risk score was then identified by the “Maximally Selected Rank Statistics” (maxstat) model (Laska et al., 2012). Then, the patients were divided into low/high risk groups based on the risk score, and the survival analysis was performed to determine the prognosis of the two groups.

### FOXF2-miRNAs and Target Genes Prediction Network

The inclusion criteria for FOXF2-miRNAs-target gene regulation network are as follows: 1) TransmiR v2.0 database (Tong et al., 2019) (http://www.cuilab.cn/transmir) is a database that provides information on the regulatory relationships between TFs and miRNAs. Thus, we first obtained the miRNAs that could be transcriptionally regulated by FOXF2; 2) According to the cutoff criteria of FC > 2.0 and FDR <0.05, the upregulated miRNAs in GC were identified by analyzing the TCGA Stomach Adenocarcinoma mature miRNA expression profile; 3) Pearson’s correlation coefficient (R value) for association between FOXF2 and miRNAs is less than −0.3; and 4) Upregulated miRNAs (FC > 2.0 and FDR <0.05) in blood of patients with GC determined by analyzing the blood miRNA expression profile from the BBCancer database (Zuo et al., 2020). The miRNAs that met the four abovementioned criteria were considered to be FOXF2 transcriptional regulated miRNAs. Then, Starbase v2.0 (Li et al., 2014) (http://starbase.sysu.edu.cn/) was used to predict the target genes of miRNAs. The predicted target genes that are downregulated in GC were included for regulation network construction.

### Gene Function Enrichment Analysis

To determine the biological function of these target genes, we performed biological pathway analysis by FunRich, which is a functional enrichment and interaction network analysis tool (Pathan et al., 2015). The pathway items with *p* < 0.05 were considered to be statistically significant.

### Statistical Analyses

All statistical analyses were performed with SPSS version 19.0 (Chicago, IL, United States) or GraphPad Prism software version 7 (GraphPad Software Inc., CA, United States). The statistical significance between two groups was analyzed by paired or unpaired t test. The Kaplan-Meier curve was analyzed by the log-rank test. Receiver operating characteristic (ROC) curves were used to test the diagnostic ability of blood miRNAs in GC. *p* < 0.05 was considered to be statistically significant.

## Results

### FOXF2 is Downregulated in GC Cells and Tissues

**FIGURE 1:**
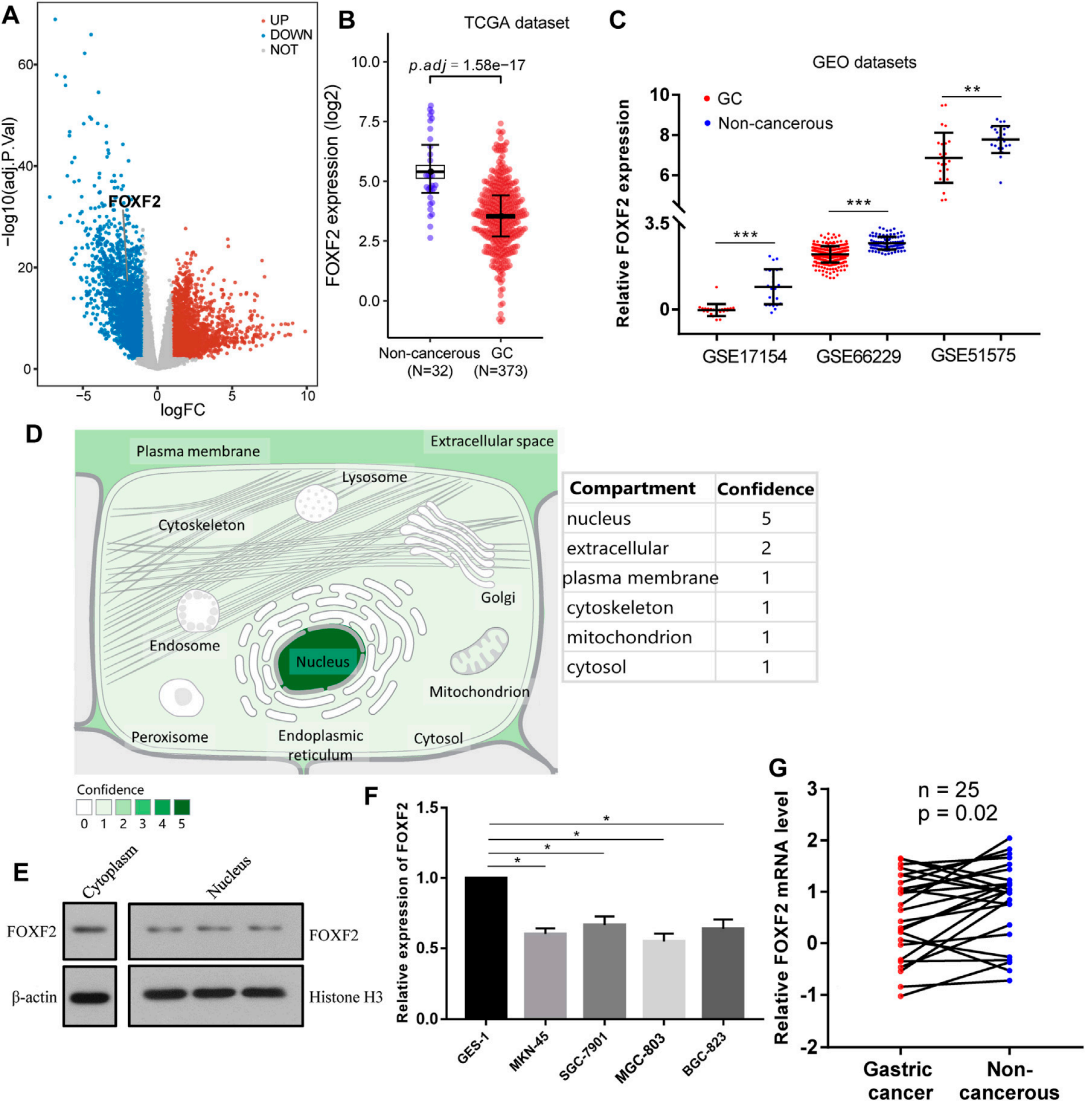
FOXF2 was downregulated in GC cells and tissues. **(A)** A total of 5,588 differentially expressed genes (2,878 highly and 2,710 low expressed genes) were identified by analyzing GC RNA-Seq data from the TCGA dataset. **(B)** According to the TCGA dataset, FOXF2 was significantly downregulated in 373 GC tissues. **(C)** Three expression profiling data from GEO datasets showed that FOXF2 was down-regulated in GC tissues. **(D)** The subcellular location prediction from COMPARTMENTS showed that FOXF2 has the greatest possibility of mainly being located in the nucleus, cytoplasm and extracellular. **(E)** The cytoplasm and nucleus protein separation assay found that FOXF2 protein was both detected in cytoplasm and nucleus. **(F,G)** The qRT-PCR assay indicated that FOXF2 was significantly downregulated in GC cells and tissues. *p < 0.05, **p < 0.01, and ***p < 0.001.

**FIGURE 2:**
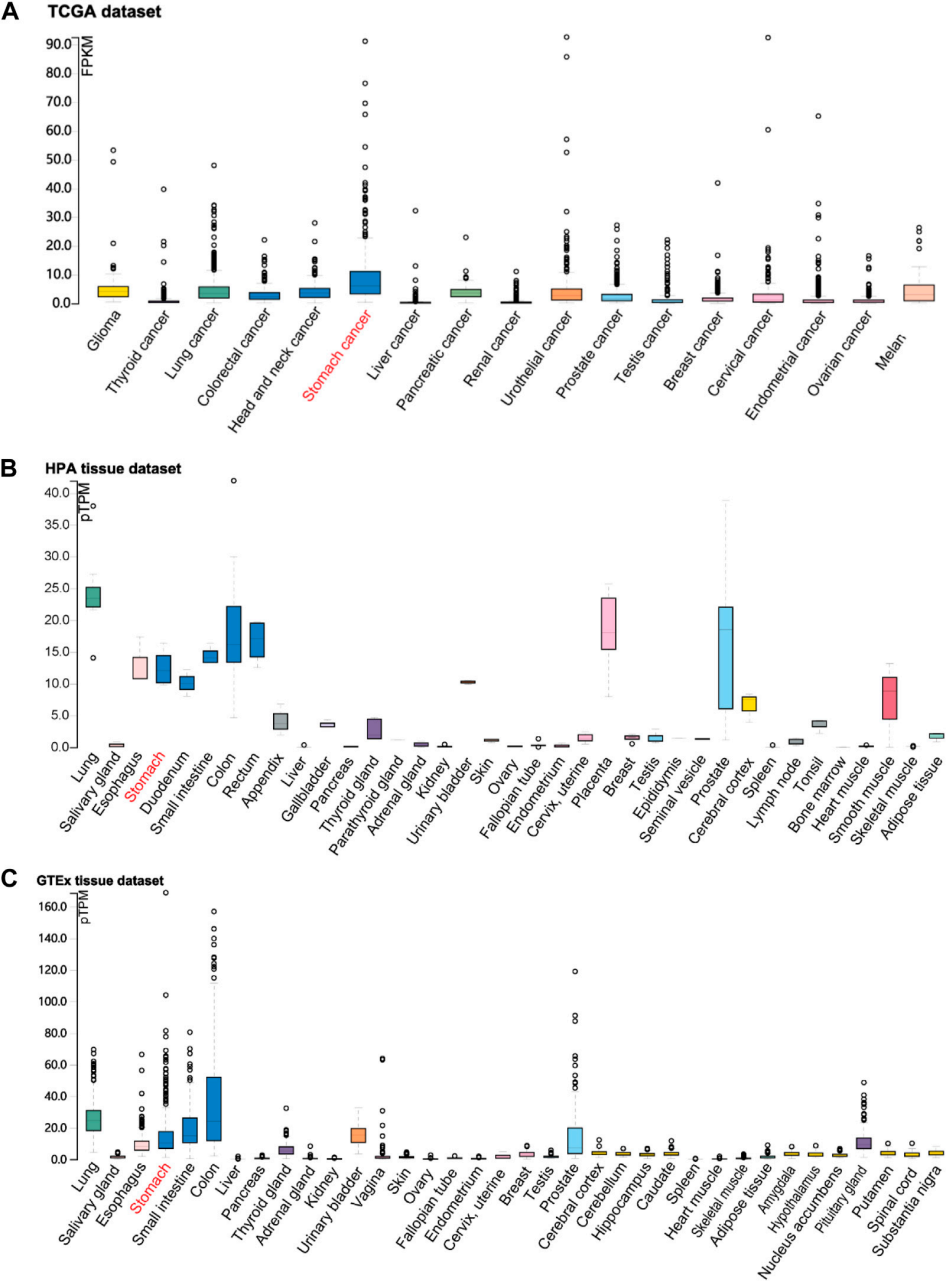
The visualization of FOXF2 expression in human tissues. FOXF2 expression level in human cancer tissues and normal tissues from TCGA **(A)**, HPA **(B)**, and GTEx **(C)** datasets.

According to the identification of DEGs, there were 2,878 highly expressed genes and 2,710 low expressed genes in GC (Figure 1A). We then analyzed 373 cases of GC tissues and 32 cases of normal gastric tissues from the TCGA database. Compared to normal tissues, the expression of FOXF2 in GC tissues was significantly decreased (Figure 1B). Three expression profiling data from GEO datasets also showed that FOXF2 was down-regulated in GC tissues (Figure 1C). The subcellular locations prediction from COMPARTMENTS (Binder et al., 2014) showed that FOXF2 was mainly located in the nucleus, cytoplasm and extracellular. (Figure 1D). Cytoplasm and nucleus protein separation assay found that FOXF2 protein was both detected in cytoplasm and nucleus (Figure 1E). Moreover, by performing qPCR on four types of GC cells, namely MKN-45, SGC-7901, MGC-803, BGC-823, and the normal gastric tissue cell line GES-1 and 25 pairs of GC tissues and adjacent normal tissues, we determined that the expression of FOXF2 is significantly downregulated in GC cells and tissues (Figures 1F,G). By using the HPA, TCGA, and GTEx databases, we analyzed the expression level of FOXF2 in various human tumor tissues and normal tissues. The results indicated that FOXF2 had quite lower expression abundance in tumor tissues and its expression varied in different types of tissues (Figures 2A–C).

### FOXF2 Dysregulation is Accompanied by Aberrant DNA Methylation

**FIGURE 3:**
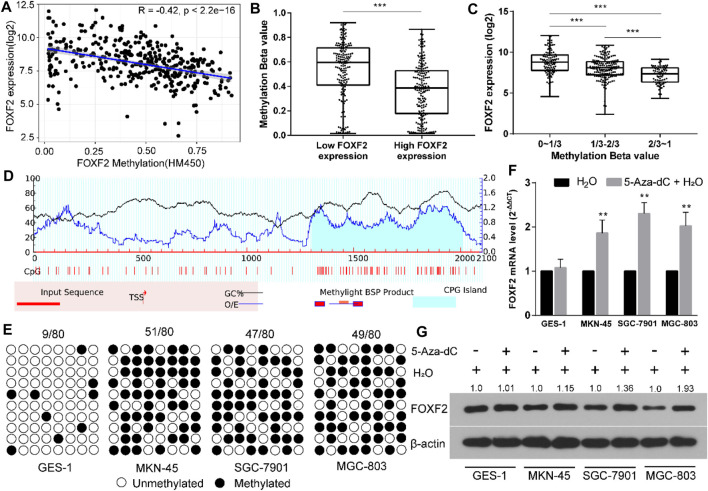
FOXF2 was downregulated through aberrant DNA methylation in the promoter region. **(A)** FOXF2 expression was negatively correlated with FOXF2 methylation level (Pearson R = −0.42, *p* < 2.2e^−16^). **(B)** A total of 372 GC samples were divided into low or high FOXF2 expression group according to the median value of FOXF2 expression. The unpaired *t*-test suggested that the FOXF2 methylation level in the high FOXF2 expression group was lower than that in the low FOXF2 expression group. **(C)** The 372 GC samples were divided into low (0–1/3), median (1/3–2/3), and high (2/3–1) methylation groups according to the tertile of the methylation level. The results showed that FOXF2 expression was lowest in the high methylation group and the highest in the low methylation group. **(D)** A CpG island with the length of 708 bp was found in the promoter region of FOXF2 by Methprimer 2.0 prediction. **(E)** The BSP assay suggested that the methylation level of the FOXF2 promoter region in GC cell lines (MKN-45, SGC-7901, and MGC-803) was higher than that in the normal control GES-1 cell line. **(F and G)** qRT-PCR and western blot assay showed that the mRNA and protein levels of FOXF2 were increased in GC cells following treatment with 5-Aza-Dc; however, no difference was found in GES-1 cells. *p < 0.05, **p < 0.01, and ***p < 0.001.

By analyzing TCGA RNA-Seq and Illumina methylation HM450 data, there are a total of 372 GC samples with valid FOXF2 expression and methylation beta data. Pearson analysis found that FOXF2 expression was significant negatively correlated with the FOXF2 methylation level (Figure 3A, Pearson R = −0.42, *p* < 2.2e-16). According to the median value of FOXF2 expression, 372 GC samples were divided into low or high FOXF2 expression group. The unpaired *t*-test indicated that the FOXF2 methylation level in the high FOXF2 expression group was lower than that in the low FOXF2 expression group (Figure 3B). We then divided the 372 GC samples into low (0–1/3), median (1/3–2/3), and high (2/3–1) methylation groups according to the tertile of the methylation level. The results showed that FOXF2 expression was the lowest in the high methylation group and the highest in the low methylation group (Figure 3C). Thus, we speculated that FOXF2 may be misregulated by aberrant DNA methylation.

The sequence from upstream −2,000 to downstream +100 bp relative to the transcription start site (TSS) of FOXF2 was regarded as the promoter region. Methprimer 2.0 predicted a CpG island (708 bp) in the promoter region of FOXF2 (Figure 3D). The methylation level of the FOXF2 promoter region in GES-1 and 3 GC cell lines was measured by the BSP assay. The results suggested that the methylation level of the FOXF2 promoter region in GC cell lines (MKN-45, SGC-7901, and MGC-803) was higher than that in the normal control GES-1 cell line (Figure 3E). These cells were then treated with the demethylation reagent 5-Aza-dC to assess the effect of methylation level on FOXF2 expression. qRT-PCR and western blot assay showed that the mRNA and protein levels of FOXF2 were increased in GC cells following treatment with 5-Aza-Dc; however, the difference was not found in GES-1 cells (Figures 3F,G).

### FOXF2 is a Prognostic index for Patients With GC

**FIGURE 4:**
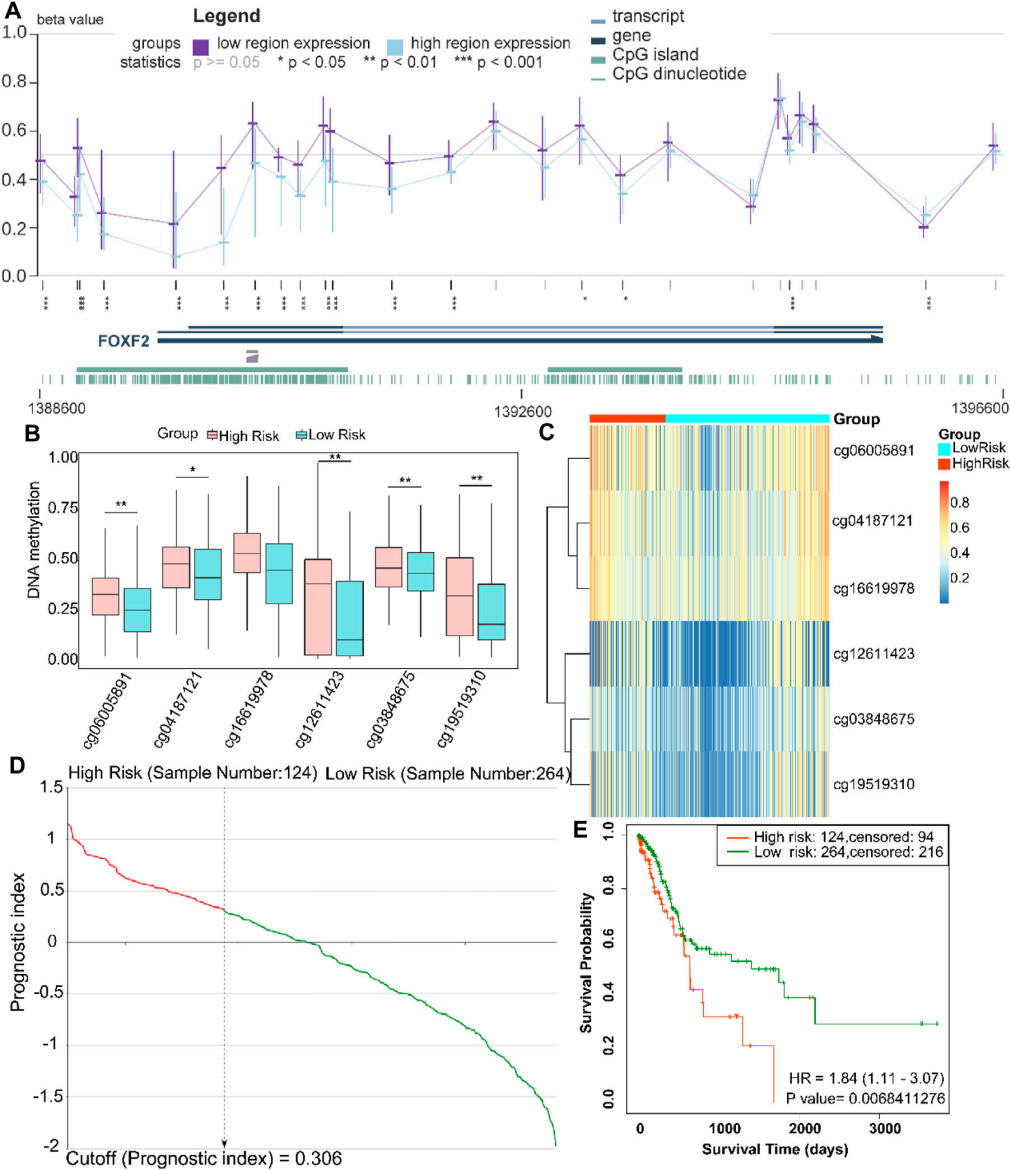
FOXF2 is a prognostic index for patients with GC patients. **(A)** The methylation levels of most of the cg loci in the low FOXF2 expression group were significantly higher than those in the high FOXF2 expression group as determined from MEXPRESS. **(B,C)** SurvivalMeth data showed that the methylation levels of 5 promoter region cg loci (cg06005891, cg04187121, cg12611423, cg03848675, and cg19519310) in the high risk group were significantly higher than those in the low risk group. The methylation level of cg16619978 in the high risk group was also higher than that in the low risk group; however, the difference was not significant. **(D)** At the optimal cutoff prognostic index of 0.306, a total of 388 GC samples were divided into high or low risk group. **(E)** The Kaplan-Meier analysis showed that patients in the high risk group had a poorer survival probability than those in the low risk group (HR = 1.84 (1.11–3.07) and *p* = 0.0068). *p < 0.05, **p < 0.01, and ***p < 0.001.

To further explore the methylation status of the FOXF2 cg locus, we analyzed the association between the methylation level of the target cg locus and FOXF2 expression using the MEXPRESS tool. It was found that the methylation levels of most of the cg loci in the low FOXF2 expression group were significantly higher than those in the high FOXF2 expression group (Figure 4A). A total of 6 cg loci located at the FOXF2 promoter region were identified by the SurvivalMeth web server. The detailed information of the cg locus is shown in Table 1. SurvivalMeth showed that the methylation levels of 5 cg loci (cg06005891, cg04187121, cg12611423, cg03848675, and cg19519310) in the high risk group were significantly higher than those in the low risk group (Figures 4B,C). Although the difference for cg16619978 was found to be nonsignificant, its methylation level in the high risk group was also higher than that in the low risk group. According to SurvivalMeth, a total of 388 GC samples were divided into a high or low risk group at the optimal cutoff prognostic index (Figure 4D). The Kaplan-Meier analysis showed that patients in the high risk group had a poorer survival probability than those in the low risk group (Figure 4E, HR = 1.84 (1.11–3.07) and *p* = 0.0068). All these findings indicated that FOXF2 can be a promising prognostic index in GC.

**TABLE 1:** Information on the 6 cg loci in the FOXF2 promoter region.

Cg locus	Chromosome	Location	Gene location	Island
cg03848675	6	1389146	TSS1500	Island
cg04187121	6	1388858	TSS1500	N shore
cg06005891	6	1389167	TSS1500	Island
cg12611423	6	1389966	TSS200	Island
cg16619978	6	1388806	TSS1500	N shore
cg19519310	6	1389367	TSS1500	Island

### FOXF2-miRNAs-Target Genes Regulation Network Construction

**FIGURE 5:**
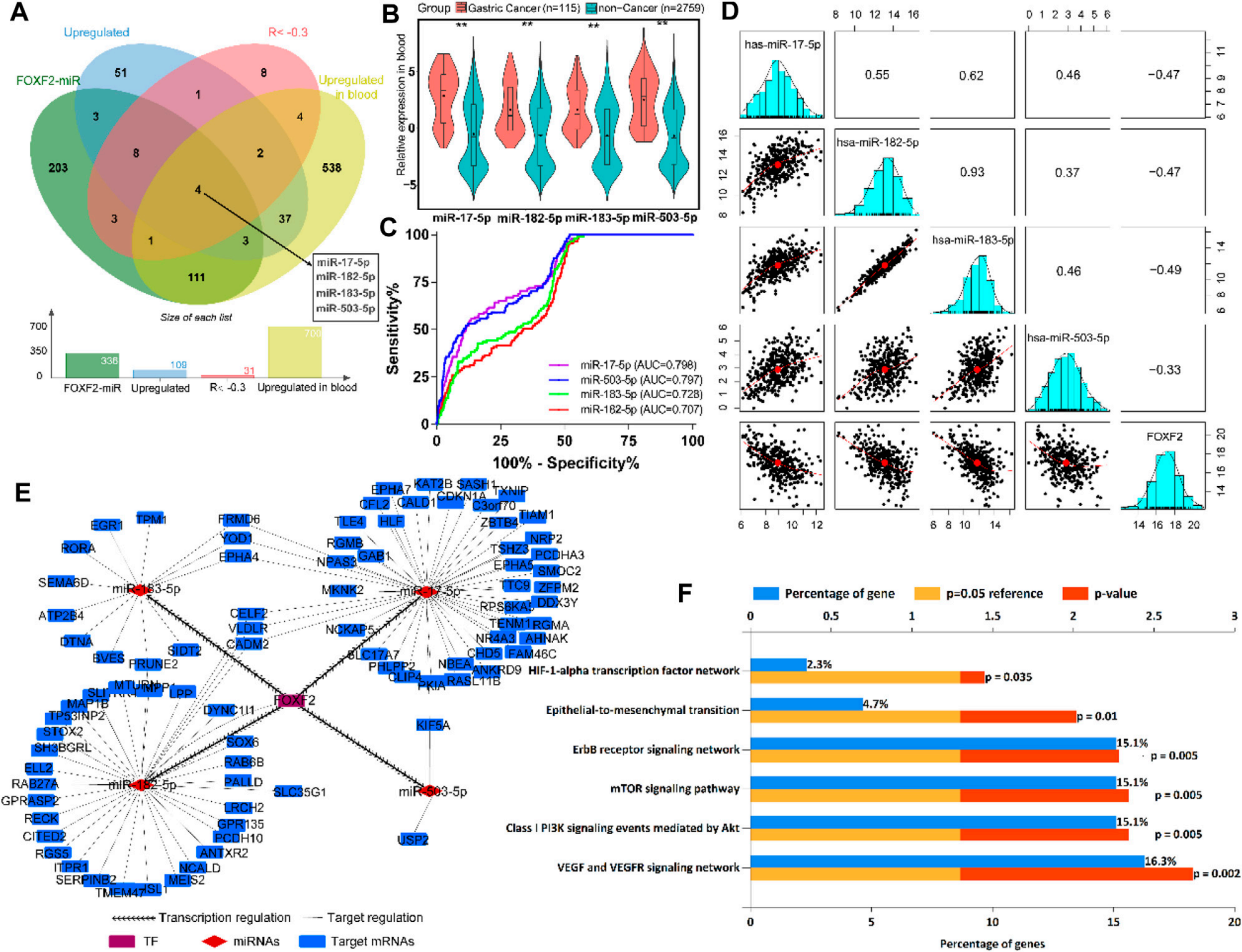
FOXF2-miRNAs-target genes regulation network construction. **(A)** Four upregulated target miRNAs (miR-17-5p, miR-182-5p, miR-183-5p, and miR-503-5p) were identified to be transrepressed by FOXF2. **(B)** The four miRNAs are significantly upregulated in the blood of patients with GC than in normal people. **(C)** ROC analysis showed that the four miRNAs have preferable ability in distinguishing patients with GC from normal people, with AUC values of more than 0.7. **(D)** Pearson’s analysis showed that FOXF2 expression was significantly negatively correlated with miR-17-5p, miR-182-5p, miR-183-5p, and miR-503-5 expression. Values in the box represent Pearson R. **(E)** FOXF2-miRNAs-target genes regulation network showed that FOXF2 had a close regulation relationship with the miRNAs and the corresponding target genes. **(F)** Target genes functional enrichment analysis found that these genes are significantly related to HIF-1α, ErbB, mTOR, epithelial-to-mesenchymal transition (EMT), Akt, VEGF, and VEGFR-mediated signaling pathway. *p < 0.05, **p < 0.01, and ***p < 0.001.

According to the previous inclusion criteria, we obtained four potential target miRNAs, including miR-17-5p, miR-182-5p, miR-183-5p, and miR-503-5p (Figure 5A). We found that compared to normal people without cancer, patients with GC showed significant upregulation of these four miRNAs in their blood (Figure 5B), which aroused our interest. ROC analysis showed that these four miRNAs had preferable ability in distinguishing patients with GC from normal individuals. The area under the curve (AUC) values were greater than 0.7, which indicated that the four miRNAs could be used as biomarkers for the diagnosis of GC (Figure 5C). Pearson’s correlation analysis showed that FOXF2 expression was significantly negatively correlated with miR-17-5p, miR-182-5p, miR-183-5p, and miR-503-5 expression (Figure 5D). Next, FOXF2-miRNAs-target genes regulation network was constructed, and it showed that FOXF2 had a close regulation relationship with the miRNAs and the corresponding target genes (Figure 5E). We then performed functional enrichment analysis of these 95 genes and found that these genes are significantly related to many important signaling pathways, including HIF-1α, ErbB, mTOR, EMT, Akt, VEGF, and VEGFR-mediated signaling pathway (Figure 5F).

## Discussion

It is known that epigenetics plays an indispensable role in the complex molecular mechanism of GC occurrence and development (Chia and Tan, 2016; Huang et al., 2018). Our team has conducted many studies on this topic (Zhang et al., 2019; Liang et al., 2020). In the present study, by exploring public databases, we found that FOXF2 was expressed to varying degrees in a variety of normal and cancer tissues in the human body. Compared to the normal gastric tissue, FOXF2 expression was significantly downregulated in GC tissues. We confirmed this through qRT-PCR assay at the cell and tissue level. In addition, we found that the downregulation of FOXF2 in GC may be due to abnormal methylation of the promoter region of FOXF2, and the methylation of the FOXF2 promoter was related to the prognosis of patients.

Many studies have shown that FOXF2 was dysregulated in different types of cancers and can be regulated by aberrant DNA methylation (Tian et al., 2015; Chen et al., 2017). FOXF2 was shown to be downregulated in GC cell lines and tissues. However, its dysregulation mechanism in GC has not yet been fully elucidated. First, we found that FOXF2 expression was significantly negatively correlated with the FOXF2 methylation level. There was also a CpG island in the promoter region of FOXF2. Thus, we speculated that FOXF2 may be regulated by aberrant DNA methylation. The BSP assay suggested that the methylation level of the FOXF2 promoter region in GC cell lines was higher than that in the normal GES-1 cell line. The cells were then treated with 5-Aza-Dc to investigate the expression change of FOXF2 induced by methylation. qRT-PCR and western blot assay revealed that the mRNA and protein levels of FOXF2 were increased in GC cells following treatment with 5-Aza-Dc. The demethylated drug 5-Aza-Dc could decrease the aberrant DNA methylation status in the gene promoter region, thus improving FOXF2 expression.

FOXF2, as a transcriptional regulator, was also found to have a transcriptional repression effect on the miR-200b ∼ 200a ∼ 429 locus in lung cancer (Kundu et al., 2016). Lu et al. (2020) also reported that FOXF2 can negatively regulate the transcription of TGF-β isoforms (TGFB1, TGFB2, and TGFB3) in basal-like breast cancer. Thus, FOXF2 may play a tumor suppressor role through transrepressing gene expression. By integrating the miRNA expression profile and TF-miRNA regulation from the TransmiR database, four miRNAs (miR-17-5p, miR-182-5p, miR-183-5p, and miR-503-5p) were identified to be the transcriptional regulation targets of FOXF2. These four miRNAs were also significantly upregulated in the blood of patients with GC as compared to that in normal people. The blood miRNA expression profile from the BBCancer database contains 115 GC and 2,759 non-cancer samples, which makes the ROC findings more reliable. Accumulating evidences revealed that the four miRNAs can play vital roles in GC tumorigenesis and development (Lin et al., 2019; Yuan et al., 2019; Huang et al., 2020; Song et al., 2020; Zhang et al., 2020). However, the expression condition in GC patient blood and dysregulated mechanism in transcriptional regulation are still not completely understood. Our findings provide evidence for studying the transcription regulation of theses miRNAs by FOXF2. As expected, these four target genes could be used as biological indicators for clinical diagnosis.

The dysregulation of FOXF2 also plays an indispensable role in tumorigenesis and development of other types of cancer. Hauptman et al. found that FOXF2 was highly methylated and downregulated in colorectal cancer, which can be used as a biomarker for the diagnosis of colorectal cancer (Hauptman et al., 2019). Zhang and Chen et al. found that miR-182 and miR-130a were highly expressed in colorectal cancer and can decrease the target FOXF2 expression, thereby promoting the proliferation, invasion, and metastasis of colorectal cancer (Zhang et al., 2015; Chen et al., 2017). Kong et al. (2016) found that FOXF2 was downregulated in non-small cell lung cancer, and the expression of FOXF2 was related to the prognosis of patients. Lo et al. (2016) found that FOXF2 may promote or suppress breast cancer tumorigenesis according to different tumor subtypes. Dou et al. (2017) showed that the downregulation of FOFX2 expression in liver cancer cells increased E-cadherin, decreased vimentin, and induced mesenchymal-epithelial transformation of liver cancer cells, thereby inhibiting their invasion and migration, but it promoted the proliferation of liver cancer cells. Hirata et al. (2013) reported that the expression of FOXF2 increased after knockdown of miR-182-5p, which can inhibit the proliferation, invasion, and metastasis of prostate cancer. In the present study, we found that FOXF2 was downregulated due to aberrant DNA methylation in GC, and the prognosis of patients with GC was related to the degree of methylation in the FOXF2 promoter region.

Our present study had some limitations. Although we verified that FOXF2 was highly correlated with four miRNAs, further experiments to verify the targeting relationship between FOXF2 and the four miRNAs were lacking. In addition, as a biological indicator of GC diagnosis, the four miRNAs need to be further verified clinically. The signaling pathways and functions of FOXF2 in GC also require further experiments to verify. The present study provides a clear direction for our future studies and could assist research on the role of FOXF2 in GC.

In conclusion, we found that FOXF2 was downregulated due to aberrant DNA methylation in GC and that the degree of methylation in the promoter region of FOXF2 was related to the prognosis of patients. Moreover, the four miRNAs, which could be transrepressed by FOXF2, may be used as biological indicators for the diagnosis of GC. These findings showed that FOXF2 can be a useful target in revealing the tumorigenesis process of GC. The methylation of FOXF2 can also serve as a prognostic risk factor for GC.

## Data Availability

Publicly available datasets were analyzed in this study. This data can be found here: RNA-Seq and DNA methylation HM450 data were downloaded from the TCGA (https://portal.gdc.cancer.gov/), GTEx (https://www.gtexportal.org/home/), HPA (https://www.proteinatlas.org/) database, Gene Expression Omnibus (accession numbers: GSE17154, GSE66229 and GSE51575) and cBioPortal database (http://www.cbioportal.org/), respectively.

## Supplementary Material

Supplementary Table S1 | Primers sequences used in present study.
