# Supplementary material for: Aberrant DNA Methylation-Mediated FOXF2 Dysregulation Is a Prognostic Risk Factor for Gastric Cancer
Source: Front Mol Biosci. 2021 Sep 10;8:645470. doi: 10.3389/fmolb.2021.645470 (PMC8460759; doi:10.3389/fmolb.2021.645470)
Supplement: Supplementary file 1 [file Table1.docx]

**Supplemental Table 1 Primers sequences used in present study.**

| **Name** | Sequence (5’-3’) |
| --- | --- |
| **PCR** |  |
| FOXF2-F | TACTCCAGTGTGTGACAGAAAA |
| FOXF2-R | TGGTGATGGTGATAATACGACC |
| GAPDH-F | AGCCACATCGCTCAGACAC |
| GAPDH-R | GCCCAATACGACCAAATCC |
| **BSP** |  |
| FOXF2-BSP-F | GGGAAGAAGTGGAAGTAAATGTAATT |
| FOXF2-BSP-R | TTTCTAACAAAACCTACAAACTCCC |
